# Lung cancer tumorigenicity and drug resistance are maintained through ALDH^hi^CD44^hi^ tumor initiating cells

**DOI:** 10.18632/oncotarget.1246

**Published:** 2013-08-30

**Authors:** Jing Liu, Zhijie Xiao, Sunny Kit-Man Wong, Vicky Pui-Chi Tin, Ka-Yan Ho, Junwen Wang, Mai-Har Sham, Maria Pik Wong

**Affiliations:** ^1^ Department of Pathology, Li Ka Shing Faculty of Medicine, The University of Hong Kong, Hong Kong SAR China; ^2^ Department of Biochemistry, Li Ka Shing Faculty of Medicine, The University of Hong Kong, Hong Kong SAR China

**Keywords:** lung cancer, tumor initiating cell, aldehyde dehydrogenase, CD44, drug resistance

## Abstract

Limited improvement in long term survival of lung cancer patients has been achieved by conventional chemotherapy or targeted therapy. To explore the potentials of tumor initiating cells (TIC)-directed therapy, it is essential to identify the cell targets and understand their maintenance mechanisms. We have analyzed the performance of ALDH/CD44 co-expression as TIC markers and treatment targets of lung cancer using well-validated *in vitro* and *in vivo* analyses in multiple established and patient-derived lung cancer cells. The ALDH^hi^CD44^hi^ subset showed the highest enhancement of stem cell phenotypic properties compared to ALDH^hi^CD44^lo^, ALDH^lo^CD44^hi^, ALDH^lo^CD44^lo^ cells and unsorted controls. They showed higher invasion capacities, pluripotency genes and epithelial-mesenchymal transition transcription factors expression, lower intercellular adhesion protein expression and higher G2/M phase cell cycle fraction. In immunosuppressed mice, the ALDH^hi^CD44^hi^ xenografts showed the highest tumor induction frequency, serial transplantability, shortest latency, largest volume and highest growth rates. Inhibition of sonic Hedgehog and Notch developmental pathways reduced ALDH^+^CD44^+^ compartment. Chemotherapy and targeted therapy resulted in higher ALDH^hi^CD44^hi^ subset viability and ALDH^lo^CD44^lo^ subset apoptosis fraction. ALDH inhibition and CD44 knockdown led to reduced stemness gene expression and sensitization to drug treatment. In accordance, clinical lung cancers containing a higher abundance of ALDH and CD44-coexpressing cells was associated with lower recurrence-free survival. Together, results suggested the ALDH^hi^CD44^hi^ compartment was the cellular mediator of tumorigenicity and drug resistance. Further investigation of the regulatory mechanisms underlying ALDH^hi^CD44^hi^ TIC maintenance would be beneficial for the development of long term lung cancer control.

## INTRODUCTION

Lung cancer is the most common malignancy but curative therapy for metastatic disease is limited. Although tyrosine kinase inhibitor (TKI) therapy achieves a higher objective response rate and longer progression free survival in cancers with epidermal growth factor receptor (*EGFR*) mutations or anaplastic lymphoma kinase (*ALK*)*-*rearrangement, relapse is the rule after 10-14 months due to drug resistance [1-5]. Increasing evidences have shown in many cancers, enhanced tumorigenicity resides in a tumor cell population that exhibits stem cell-like properties such as self-renewal, differentiation, cell mobility and toxicity resistance, designated as cancer stem cells (CSC) or tumor initiating cells (TIC) [6, 7]. Inhibition of morphogenesis regulatory pathways such the Hedgehog, Notch and Wnt/β-catenin pathways can retard tumor transplantability, further supporting TIC-targeting could be useful for lung cancer control [8-11]. However, a deeper understanding of the cell targets and maintenance mechanisms is essential for further therapy development.

Lung cancer cells expressing various molecules such as CD133, CD166, ALDH, CXCR4, GLDC, etc. have been shown to demonstrate TIC phenotypic characteristics [12-15]. On the other hand, to identify TIC with higher specificity, more stringent selection strategies such as the adoption of co-expressed markers and verification in larger numbers of cell lines including patient-derived samples are advocated. We have previously demonstrated lung cancer cells with high CD44 expression were enriched for stem cell-like properties [16]. Moreover, CD44 is expressed in breast, colon and gastric cancer stem cells [17-19]. Aldehyde dehydrogenase 1 (ALDH) is expressed in murine embryonic lungs and has been reported to select for human lung TIC [15, 20, 21]. The specificity of combined ALDH/CD44 expression as lung TIC marker compared to either marker alone is not known. In this study, we have used a range of established and patient-derived lung cancer cell lines (PDCL) to show cells with high ALDH and CD44 co-expression (ALDH^hi^CD44^hi^) possessed *in vitro* and *in vivo* TIC properties with enhanced tumorigenicity and drug resistance compared to the low-expressing (ALDH^lo^CD44^lo^) compartment or un-selected cells. Simultaneous ALDH inhibition and CD44 depletion as well as pharmacologic inhibition of Hedgehog or Notch attenuated TIC characteristics. In clinical lung cancers, recurrence-free survival was longer for patients with low abundance ALDH/CD44-coexpressing cells (*p* = 0.053). Our data demonstrated lung TIC are enhanced through ALDH and CD44 co-regulating pathways. Further investigation of the ALDH^hi^CD44^hi^ population would enable a better understanding of TIC regulation and facilitate development of therapeutic strategies for long term lung cancer control.

## RESULTS

### ALDH^hi^CD44^hi^ population displayed *in vitro* TIC properties

**Figure 1:**
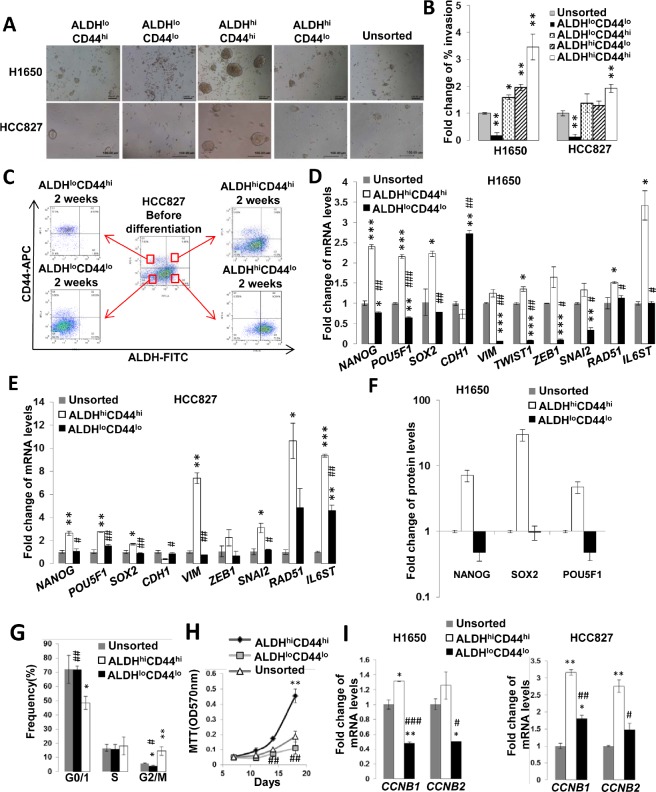
ALDH^hi^CD44^hi^ lung cancer cells showed *in vitro* TIC characteristics A, Spheroid formation assay. FACS-isolated lung cancer cell populations with differential ALDH/CD44 expressions and unsorted cell controls were kept in serum-free non-adherent plates for 21 days. B, Matrigel invasion assay. The proportions of invading cells from respective cell subsets were normalized to the unsorted control. C, *In vitro* differentiation assay. The 4 freshly isolated populations were separately cultured in adhesive plates containing normal medium for 2 weeks. Cells were then freshly harvested and re-analyzed by flow cytometry for ALDH/CD44 expression profile. The central profile represented parental unsorted cells and profiles of the subsets were as labeled. D and E, Normalized mRNA expressions of pluripotency, EMT and other genes by QPCR. F, Pluripotency proteins expression analyzed by flow cytometry. Results were normalized to unsorted control. G, Cell cycle analysis. Freshly isolated ALDH^hi^CD44^hi^ and ALDH^lo^CD44^lo^ populations of H1650 were stained with propidium iodide and analyzed by flow cytometry for DNA content. H, Cell proliferation assay. Respective subsets of freshly isolated H1650 cells were analyzed by MTT. I, Expression of *CCNB1* and *CCNB2* analyzed by QPCR.*, *p* < 0.05; **, *p* < 0.01; ***, *p* < 0.001, compared with unsorted; #, *p* < 0.05; ##, *p* < 0.01; ###, *p* < 0.001, compared with ALDH^hi^CD44^hi^. All data represent the mean ± SD of triplicate experiments.

The ALDH/CD44 co-expression profiles of 11 lung cancer cell lines including PDCL and drug-induced resistant cells were analyzed by flow cytometry. In 6 cell lines, ALDH/CD44 co-expressing cells (ALDH^+^/CD44^+^) comprised the smallest subset with ALDH/CD44 non-expressing cells (ALDH^−^/CD44^−^) forming the largest population (Supplementary Table S1). Subsequently, the top/bottom 1 to 5% of cells showing highest/lowest expression of the markers (ALDH^hi^CD44^hi^, ALDH^hi^CD44^lo^, ALDH^lo^CD44^hi^ and ALDH^lo^CD44^lo^) were freshly isolated from H1650 and HCC827 cell lines for further in vitro tests. In the spheroid formation assay, the ALDH^hi^CD44^hi^ populations generated more abundant and larger spheroid bodies than the other 3 subsets (Figure 1A). In the cell invasion assay, they demonstrated the highest percentage of invading cells while the ALDH^lo^CD44^lo^ subset showed the lowest (Figure 1B). *In vitro* differentiation in normal culture conditions showed only the ALDH^hi^CD44^hi^ subset was able to differentiate into all 4 cell populations with similar distribution profile as the parental cell line while compositions of the other 3 subsets remained largely unchanged from their fresh, post-sorting profiles (Figure 1C).

The ALDH^hi^CD44^hi^ population showed expression profiles that were characteristic of TIC. They had significantly higher expression of the pluripotency genes *NANOG, POU5F1* and *SOX2* at both the mRNA and protein levels compared to ALDH^lo^CD44^lo^ and unsorted populations (Figure 1D to F). They also showed higher mRNA expression of the epithelial to mesenchymal transition (EMT) transcription factors *ZEB1* and *SNAIL2*, the mesenchymal gene *VIM*, the DNA double strand break repair gene *RAD51*, the TGFβ/IL6 axis gene *IL6ST*, and a lower expression level of the cell adhesion molecule *CDH1* (Figure 1D & E).

### ALDH^hi^CD44^hi^ showed G2/M shift compared to ALDH^lo^CD44^lo^ subset in cell cycle analysis

Cell cycle analysis showed the ALDH^hi^CD44^hi^ subset of H1650 had a significantly higher proportion in G2/M phase (14.57 ± 3.23%) compared to ALDH^lo^CD44^lo^ (3.74 ± 0.59%, *p* < 0.05) and unsorted controls (5.81 ± 0.23%, *p* < 0.01) while cells in G0/1 phase were less abundant (48.42 ± 4.48%) than the other populations (ALDH^lo^CD44^lo^, 71.84 ± 2.58%, *p* < 0.01; unsorted, 72.06 ± 9.98%, *p* < 0.05) (Figure 1G). Cell growth kinetics analysis showed a higher growth rate of ALDH^hi^CD44^hi^ than unsorted and ALDH^lo^CD44^lo^ cells (Figure 1H). Correspondingly, the mRNA expression of the G2/M phase cell cycle genes *CCNB1* and *CCNB2* were significantly higher in ALDH^hi^CD44^hi^ than either ALDH^lo^CD44^lo^ or unsorted cells (Figure 1I).

### ALDH^hi^CD44^hi^ subset showed the highest tumorigenicity in immunosuppressed mice

**Figure 2:**
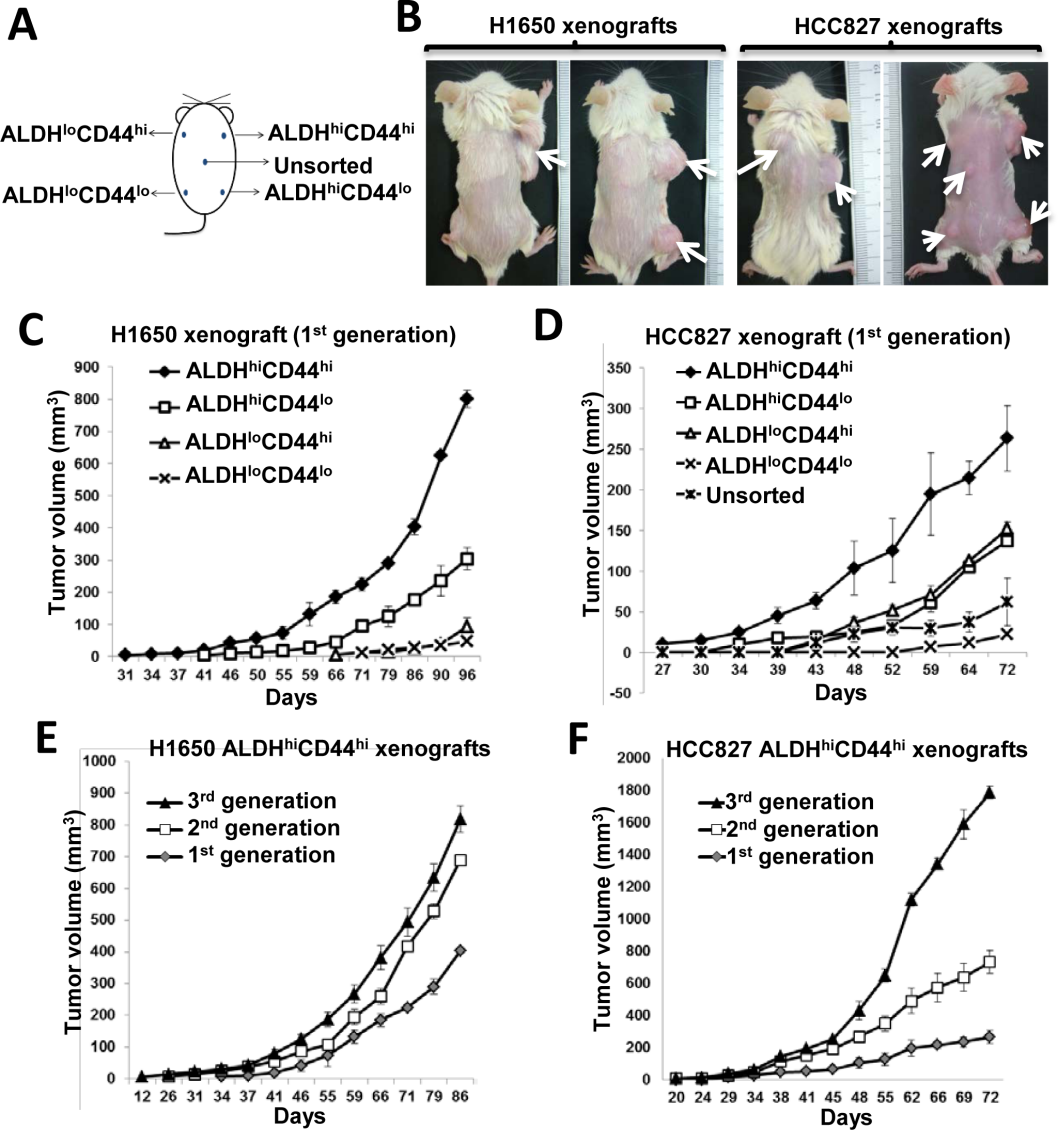
ALDH^hi^CD44^hi^ population showed TIC properties *in vivo* A, Schematic diagram showing transplantation sites of respective ALDH/CD44 populations in SCID mice. B, Representative photographs of H1650 or HCC827 xenografts derived from respective ALDH/CD44 populations after 3 months tumor development. C and D, Tumor growth curves of 1^st^ generation xenografts derived from 2,500 cells of respective ALDH/CD44 populations. E and F, tumor growth curves of 3 serially transplanted generations of xenografts derived from 2,500 ALDH^hi^CD44^hi^ cells of respective cell lines. Data represent the mean ± SD of tumor volumes at different time points of different groups, there are 6 mice in each group.

To test for *in vivo* tumorigenicity, the 4 freshly harvested ALDH/CD44 populations and unsorted controls were transplanted subcutaneously into SCID mice according to their assigned locations (Figure 2A & B) from 3 established cell lines and 4 PDCLs. As shown in Table 1, the ALDH^hi^CD44^hi^ population of all analyzed cell lines demonstrated higher graft rates, shorter latency and larger volumes of xenografts compared to the same dose of ALDH^lo^CD44^hi^, ALDH^hi^CD44^lo^ or ALDH^lo^CD44^lo^ cells (Figure 2C & D). As few as 500 ALDH^hi^CD44^hi^ cells of HCC827 or H1650 but not other populations were able to initiate tumors at this low seeding dose (Supplementary Table S2). Interestingly, the HKULC3 cell line, which did not form xenografts from 10^8^ unsorted cells transplanted in nude mice for 6 months in our previous study, was able to initiate tumor in SCID mice from 4,000 ALDH^hi^CD44^hi^ cells in 3 months ([22], Table 1).

**Table 1:** Serial transplantation of ALDH/CD44 subsets from different NSCLC cell lines

Cell line and subset	1st generation		2nd generation		3rd generation	
	Tumor incidence	Latency (days)	Tumor incidence	Latency (days)	Tumor incidence	Latency (days)
H1650	2,500 cells injection		2,500 cells injection		2,500 cells injection	
Unsorted	3/6	69.6 ± 5.6	0/5	N/A	ND	ND
ALDH^lo^CD44^lo^	2/6	75.0 ± 4.0	1/6	89	1/6	90
ALDH^lo^CD44^hi^	3/6	72.7 ± 6.7	4/6	64.0 ± 10.8	3/5	35.3 ± 1.0
ALDH^hi^CD44^lo^	4/6	43.5 ± 2.5	5/6	53.4 ± 3.8	4/5	50.8 ± 1.3
ALDH^hi^CD44^hi^	6/6	41.0 ± 5.0	6/6	25.8 ± 2.0	6/6	19.0 ± 1.8
HCC827	2,500 cells injection		2,500 cells injection		2,500 cells injection	
Unsorted	2/3	42.7 ± 2.9	3/4	60.0 ± 11.1	0/4	N/A
ALDH^lo^CD44^lo^	1/3	59	2/4	65.5 ± 4.9	0/4	N/A
ALDH^lo^CD44^hi^	2/3	41.0 ± 2.0	3/3	63.0 ± 6.9	1/4	43
ALDH^hi^CD44^lo^	2/3	41.0 ± 7.5	4/4	53.3 ± 9.5	2/4	44.5 ± 2.1
ALDH^hi^CD44^hi^	3/3	29.0 ± 1.0	4/4	22.0 ± 1.6	4/4	20.2 ± 1.8
PDCL #24	30,000 cells injection		2,500 cells injection		2,500 cells injection	
Unsorted	3/3	22.0 ± 1.2	1/3	44.0	1/3	50.0
ALDH^lo^CD44^lo^	2/3	33.5 ± 2.0	1/3	44.0	0/3	N/A
ALDH^lo^CD44^hi^	3/3	22.0 ± 1.2	3/3	34.0 ± 1.0	2/3	45.2 ± 3.7
ALDH^hi^CD44^lo^	N/A	N/A	0/2	N/A	N/A	N/A
ALDH^hi^CD44^hi^	3/3	8.7 ± 1.5	3/3	20.7 ± 2.1	3/3	18.7 ± 3.2
PDCL #2	8,000 cells injection		8,000 cells injection		ND	
Unsorted	0/3	N/A	0/3	N/A	ND	ND
ALDH^lo^CD44^lo^	0/3	N/A	0/3	N/A	ND	ND
ALDH^lo^CD44^hi^	0/3	N/A	0/3	N/A	ND	ND
ALDH^hi^vCD44^lo^	0/3	N/A	0/3	N/A	ND	ND
ALDH^hi^CD44^hi^	2/3	150.0 ± 42.4	3/3	39.0 ± 1.7	ND	ND
PDCL #10	1,500 cells injection		1,200 cells injection		600 cells injection	
Unsorted	0/3	N/A	0/3	N/A	ND	ND
ALDH^lo^CD44^lo^	0/3	N/A	0/3	N/A	ND	ND
ALDH^lo^CD44^hi^	3/3	20.0 ± 5.0	0/3	N/A	0/3	N/A
ALDH^hi^CD44^lo^	0/3	N/A	0/3	N/A	0/3	N/A
ALDH^hi^CD44^hi^	3/3	9.0 ± 2.0	3/3	12.0 ± 3.0	3/3	52.1 ± 8.5
PDCL #18	10,000 cells injection		ND		ND	
Unsorted	0/2	N/A	ND	ND	ND	ND
ALDH^lo^CD44^lo^	0/2	N/A	ND	ND	ND	ND
ALDH^lo^CD44^hi^	1/2	35.0	ND	ND	ND	ND
ALDH^hi^CD44^lo^	0/2	N/A	ND	ND	ND	ND
ALDH^hi^CD44^hi^	2/2	17.0 ± 4.0	ND	ND	ND	ND
HKULC3	4,000 cells injection		4,000 cells injection		ND	
Unsorted	0/3	N/A	ND	ND	ND	ND
ALDH^lo^CD44^lo^	0/3	N/A	ND	ND	ND	ND
ALDH^lo^CD44^hi^	0/3	N/A	ND	ND	ND	ND
ALDH^hi^CD44^lo^	0/3	N/A	ND	ND	ND	ND
ALDH^hi^CD44^hi^	3/3	92.3 ± 4.0	3/3	76.3 ± 6.9	ND	ND

N/A, tumor dimension not available; ND, not done

Serial transplantation showed ALDH^hi^CD44^hi^–derived tumors had increasing induction frequencies, reduced latency and increased growth rates in successive generations compared to the first-generation transplants (Table 1, Figure 2E & F). In contrast, the tumor frequencies of ALDH^lo^CD44^hi^, ALDH^hi^CD44^lo^, ALDH^lo^CD44^lo^ or unsorted cells decreased in the second and tertiary generations (Table 1).

### ALDH^hi^CD44^hi^ population showed *in vitro* and *in vivo* resistance to anti-cancer drugs

**Figure 3:**
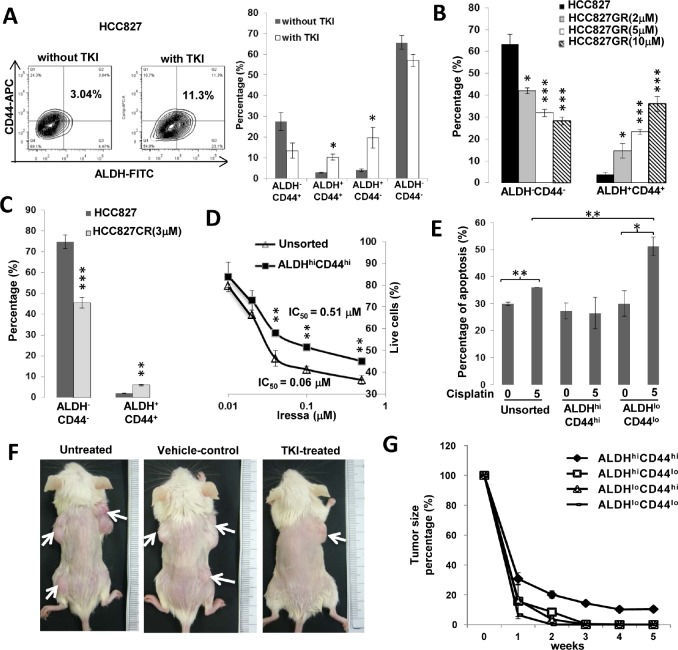
ALDH^hi^CD44^hi^ cells showed drug resistance *in vitro* and *in vivo* A, Effects of 30 nM gefitinib treatment for 24 hr on ALDH/CD44 subsets of HCC827 analyzed by flow cytometry. B and C, Proportions of ALDH^+^CD44^+^ and ALDH^−^CD44^−^ cells in drug-resistant HCC827 cells. Resistance was induced by chronic exposure to increasing gefitinib doses (HCC827-GR) or to cisplatin (HCC827-CR). D, *In vitro* cell viability response. Freshly isolated ALDH^hi^CD44^hi^ and unsorted cells of HCC827 were treated with a range of gefitinib doses for 24 hr and cell viability was assayed by MTT. E, Apoptosis response. Freshly isolated respective populations and unsorted H1650 were treated with 5 μM cisplatin for 24 hr and apoptosis fractions were assayed by Annexin V/PI staining. F, *In vivo* xenograft response after TKI treatment. Mice bearing HCC827 xenografts from respective ALDH/CD44 subsets as depicted in Figure 2A were given intraperitoneal gefitinib 5 times per week for 5 weeks. G, *In vivo* tumor response curve. Tumor volumes of respective HCC827 xenografts in TKI-treated (n = 5) or control-treated (n = 3) mice were measured twice weekly. Data represent mean ± SD of tumor volume normalized to pre-treatment size in gefitinib-treated mice. *, *p* < 0.05; **, *p* < 0.01; ***, *p* < 0.001, compared with control treatment or unsorted cells.

The response of ALDH/CD44 subsets to both targeted drugs and cytotoxic chemotherapy were compared using *in vitro* and *in vivo* models. Gefitinib treatment of the TKI-sensitive HCC827 (*EGFR* exon19 deletion) led to significant elevation of ALDH^+^CD44^+^ and ALDH^+^CD44^−^ subsets (Figure 3A). In cells induced by chronic exposure to increasing gefitinib dosage (HCC827-GR), progressive resistance was associated with stepwise increase in ALDH^+^CD44^+^ proportions (Figure 3B). This population was also higher in HCC827 induced for cisplatin-resistance (HCC827-CR) (Figure 3C). Cell viability to gefitinib treatment was significantly higher for ALDH^hi^CD44^hi^ compared to unsorted HCC827 cells (Figure 3D). Conversely, the apoptotic fractions after 24 hrs cisplatin treatment were significantly higher in ALDH^lo^CD44^lo^ (*p* < 0.05) and unsorted H1650 cells (TKI-resistant, cisplatin-sensitive) (*p* < 0.01) while no effect was observed in the ALDH^hi^CD44^hi^ population (Figure 3E). Amongst 5 TKI-treated mice bearing HCC827 xenografts, only ALDH^hi^CD44^hi^-derived tumors persisted after 4-5 weeks intraperitoneal gefitinib while no residual tumor was detected histologically at other injection sites (Figure 3F & 3G). No reduction in tumor size was observed in mice receiving control treatment.

### Hedgehog and Notch pathway inhibition suppressed ALDH^hi^CD44^hi^ and spheroid formation

**Figure 4:**
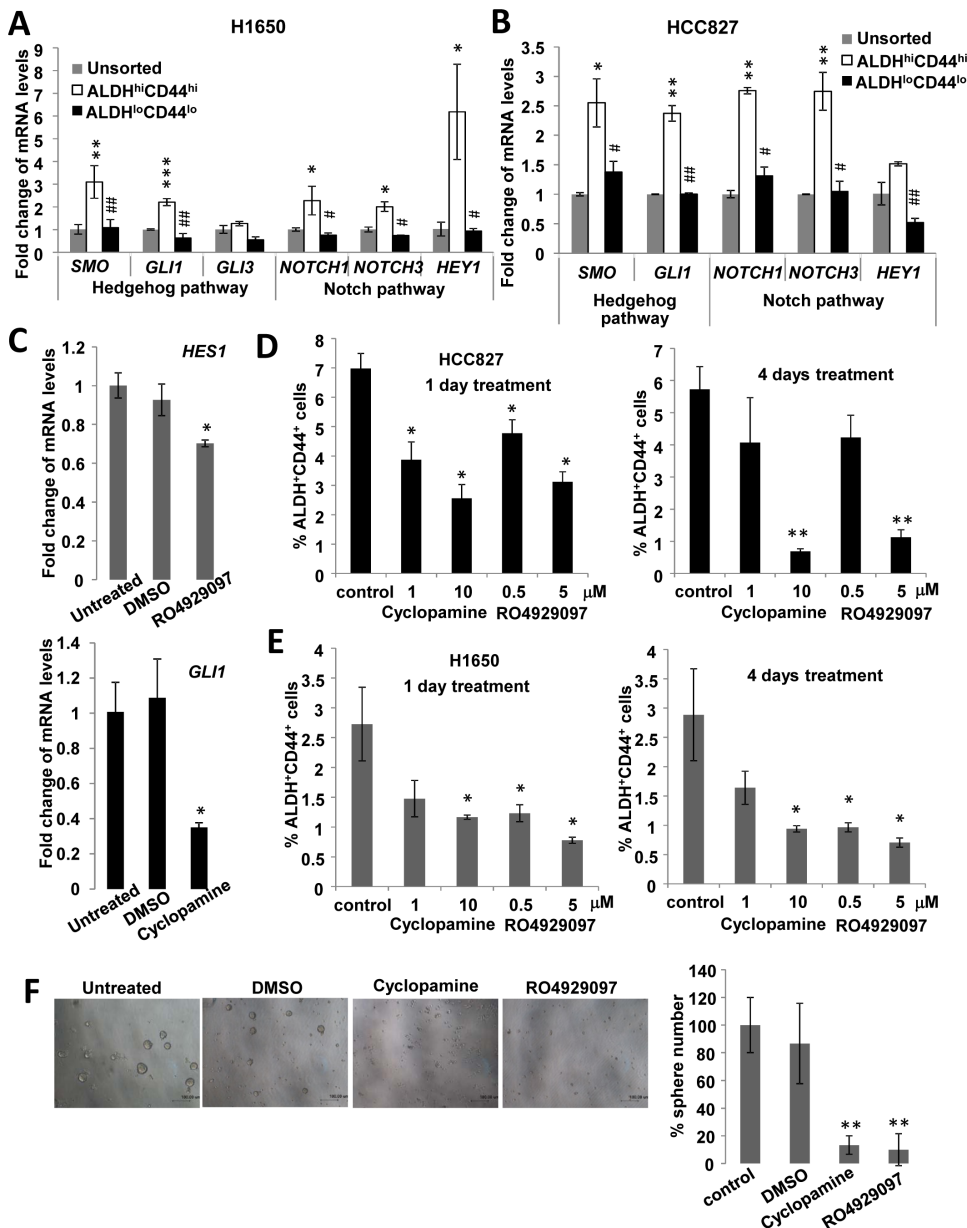
Hedgehog and Notch signaling were involved in ALDH^hi^CD44^hi^ TIC maintenance A and B, mRNA levels of Hedgehog and Notch signaling genes by QPCR in sorted cells from H1650 and HCC827. C, mRNA levels of *HES1* or *GLI1* by QPCR in HCC827 after Notch inhibition by RO4929097 or Hedgehog inhibition by cyclopamine. D and E, Proportions of ALDH^+^CD44^+^ by flow cytometry in HCC827 and H1650 after pathway inhibition for 1 or 4 days. F, Spheroid formation assay of HCC827 cells treated with inhibitors. *, p < 0.05; **, p < 0.01; ***, p < 0.001, compared with unsorted or control; #, p < 0.05; ##, p < 0.01, compared with ALDH^hi^CD44^hi^. Data represent mean ± SD of triplicate experiments.

To investigate whether morphogenesis pathways were involved in ALDH^hi^CD44^hi^ subset maintenance, the effects of pharmacological inhibition of Hedgehog and Notch pathways were analyzed. The expression of sonic Hedgehog (*SMO, GLI1*) and Notch (*NOTCH1, NOTCH3, HEY1*) pathway genes were significantly higher in ALDH^hi^CD44^hi^ compared to ALDH^lo^CD44^lo^ populations in both H1650 and HCC827 (Figure 4A & B). Cyclopamine treatment led to mRNA suppression of the Hedgehog transcription factor *GLI1* by >50% while RO4929097 suppressed the Notch transcription factor *HES1* by 30%, respectively (Figure 4C). These treatments resulted in reduction of the ALDH^+^CD44^+^ in both cell lines as well as inhibited spheroid formation, indicating their involvement in ALDH^hi^CD44^hi^ subset maintenance (Figure 4D to 4F).

### CD44 knockdown and ALDH inhibition sensitized tumor cells to in vitro anti-cancer drugs

**Figure 5:**
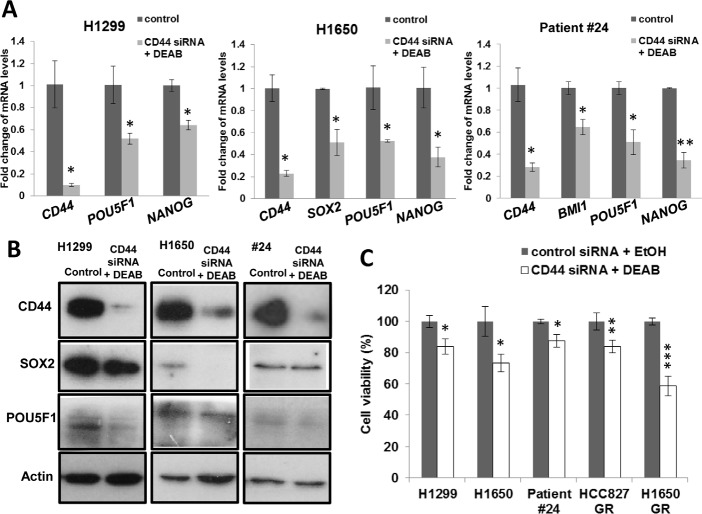
CD44 knockdown and ALDH inhibition reduced pluripotency gene expression and sensitized cells to drug treatment A and B, mRNA and protein expressions of CD44 and pluripotency genes. CD44 was depleted by siRNA and ALDH inhibited by DEAB (100 μM) in H1299, H1650 and PDCL #24, control siRNA and Ethanol were used as control. Histograms represent mean ± SD of mRNA measured by QPCR in triplicates and protein expression was analyzed by western blot. C, Cell viability assay. CD44 and ALDH-inhibited or control cells were treated respectively with cisplatin (H1650, 10 μM; H1299 & PDCL #24, 15 μM) or gefitinib (HCC827 GR, 10 μM; H1650 GR, 20 μM) for 24 hrs. *, *p* < 0.05; **, *p* < 0.01; ***, *p* < 0.001, compared with control. Data represent mean ± SD of triplicate experiments.

To investigate the role of ALDH and CD44 in TIC maintenance, ALDH enzymatic activity was completely inhibited by DEAB and CD44 was down-regulated with 80-90% reduction in mRNA and protein expression in multiple cancer cell lines (Figure 5A & B). These treatments led to a variable reduction of developmental regulatory genes expression by 30-50% including *POU5F1, NANOG, SOX2* and *BMI1* (Figure 5A & B). It also resulted in significant sensitization to cisplatin (H1299, H1650, PDCL #24, *p* < 0.05) and gefitinib treatment ((HCC827-GR, *p* < 0.01; H1650-GR, *p* < 0.001) with reduced cell viability (Figure 5C).

### Lung cancer patients with lower abundance of tumor cells co-expressing ALDH and CD44 had longer recurrence-free survival

Immunohistochemical analysis of 193 resected lung cancers of mixed histological types showed ALDH was expressed in 82 (42.5%) and CD44 in 119 (61.7%) cases, respectively (Supplementary Table S4). ALDH and CD44 expression were both associated with squamous cell carcinomas (χ2 test, *p* = 0.035 and *p* = 0.005, respectively). No association of either marker with tumor differentiation, pathological stage, patient gender or smoking history was found. There was no correlation between cases that showed ALDH and CD44 expression. Clinicopathological variables such as gender, smoking history, age, pathological stage, tumor type, differentiation, and ALDH or CD44 single marker expression were included in Cox regression multivariate analysis for recurrence-free survival (RFS), and results showed the male gender (HR 2.89, 95% CI 1.48-5.63, *p* = 0.002) and advanced pathological stage (HR 1.96, 95% CI 1.50-2.56, *p* < 0.001) were independent prognostic factors associated with shorter RFS.

**Figure 6:**
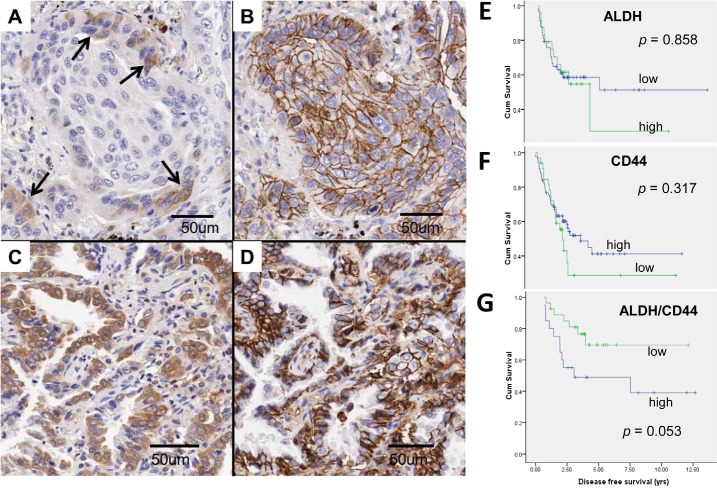
ALDH and CD44 expression in clinical lung cancers A to D, ALDH/CD44 co-expression patterns were variable in different tumors. A and B, Cytoplasmic ALDH expression (A) was observed in only a few tumor cells (→) at the invasion front of this squamous cell carcinoma while membranous CD44 expression (B) was present in all tumor cells in the same cluster. Co-localizing cells showing both ALDH and CD44 expression were few and thus graded as low abundance. C and D, This adenocarcinoma showed co-expression of ALDH (C) and CD44 (D) in almost all tumor cells and was thus graded as high abundance. E to G, Kaplan Meier survival curves comparing recurrence-free survival (RFS) in different tumor groups. For single marker analysis of ALDH (E) or CD44 (F), no significant difference in RFS was observed between tumor groups showing low or high abundance. For ALDH/CD44 dual marker analysis (G), RFS was shorter for patients with high compared to low abundance ALDH/CD44-coexpressing TIC (*p* = 0.053).

In 43 (22.3%) tumors, neither ALDH nor CD44 was expressed. In 47 (24.4%) cases, ALDH/CD44 co-expressing cells were detected, including 27 (57.4%) showing only a few scattered co-expressing cells (low abundance) and 20 (42.6%) cases that showed large aggregates or confluent sheets of such tumor cells (high abundance) (Figure 6A to D). In this group, survival analysis by log rank test showed there was no association between RFS and ALDH or CD44 single marker expression (Figure 6E & F). However, the low abundance ALDH/CD44 co-expressing group had longer RFS than the high abundance group (*p* = 0.053) (Figure 6G). The effect on overall survival did not reach statistical significance (*p* = 0.141).

## DISCUSSION

Cellular heterogeneity encompassing a TIC population that carries enhanced tumorigenic potentials has been demonstrated in many cancers but the mechanisms maintaining such populations remain unclear. To study the regulatory mechanisms, the distinction of TIC from non-TIC is required but opinions differ on the most appropriate method of TIC identification. Some investigators employ the cell spheroid as an *in vitro* functional TIC indicator but the TIC purity, particularly of large spheroids, has seldom been documented and its applicability in mechanistic studies is unclear. On the other hand, the employment of cell surface markers allows the *in vitro* isolation of a particular cell population but no single marker has been found to be universally applicable for all cancers and the specific scenario being investigated needs to be noted. In this study, we have employed a series of well-validated *in vitro*, *in vivo* and expression assays to demonstrate the association of TIC properties with the ALDH^hi^CD44^hi^ phenotype in a panel of established lung cancer cell lines as well as PDCL. Transplantation studies using multiple cell lines and PDCL consistently showed tumors derived from ALDH^hi^/CD44^hi^ cells had the highest tumor induction and growth rate, lowest seeding dose, and shortest latency in immunodeficient mice that could be serially passaged for at least 3 generations. Early passage PDCL which are generally believed to be less adapted to *in vitro* growth conditions could be serially transplanted through the ALDH^hi^CD44^hi^ subset but much less readily through the other 3 populations. Compared to CD44 or ALDH single marker, tumorigenicity was enhanced in cells selected by ALDH^hi^CD44^hi^ combined marker ([16] and Supplementary Table S3). Moreover, the ALDH^+^CD44^+^ population and cell spheres formation were inhibited by pharmacological disruption of developmental programs such as the sonic Hedgehog and Notch pathways. The results of these comprehensive assays showed under *de novo* experimental conditions, tumor cells showing the highest expression of ALDH^hi^CD44^hi^ combined markers demonstrated enhanced TIC properties. This marker combination has also been shown to associate with TIC potentials in head and neck as well as breast cancers [23, 24].

Interestingly, results of *in vitro* differentiation studies were consistent with the expected identity of ALDH^hi^CD44^hi^ population as TIC as it yielded 4 progenies distributed in proportions resembling the parental cell line while the others remained largely as the original phenotype lacking differentiation capacity. However, in the xenografts, tumors derived from all populations including ALDH^lo^CD44^lo^ showed similar ALDH/CD44 profiles (data not shown). This could be due to phenotypic plasticity with conversion of non-TIC into TIC under tumor microenvironment stimulation. Experimentally, the TGFβ/IL6 axis has been proposed to be involved in CSC maintenance. We found that exogenous TGFβ stimulation of HCC827 induced a significant increase of ALDH^+^CD44^+^ cells from 3.63% to 9.38% (*p* < 0.05) (Supplementary Figure S1). TGFβ is shown to be involved in CD44 regulation in breast CSC maintenance while interleukin 6 (IL6) can promote growth and survival of glioma stem cells [25, 26]. These data not only caution against a purely *in vitro* approach in studying TIC regulation but also indicate disrupting stroma-cancer interactions is important in anti-TIC therapy.

One of the major implications of the CSC theory is that chemotherapy or target therapy effects are mainly incident on the non-TIC thus compromising long term cancer control [27-29]. Our *in vitro* and *in vivo* results argue in favor of this hypothesis. The IC_50_ of gefitinib was almost 8.5 fold higher for TIC than unsorted cells of HCC827, while cisplatin produced higher apoptotic rate in non-TIC than TIC of H1650 (Figure 3). Only subcutaneous xenografts derived from ALDH^hi^CD44^hi^ progenies persisted after intraperitoneal gefitinib injection. The mechanism mediating the differential drug resistance is likely to be multifactorial. In both animal and human studies, ALDH mediates detoxification and cyto-protection against multiple agents such as aldehydes, alkylating drugs, oxidative stress, etc. On the other hand, through the retinoid signaling pathway, it is involved in self-renewal and tissue differentiation [30, 31]. For CD44, binding to hyaluronan increases NANOG phosphorylation and nuclear translocation, subsequently leading to the upregulation and stabilization of multidrug-resistant protein 1 (MDR1) for drug export [32]. CD44 interaction with POU5F1-SOX2-NANOG signaling also plays a pivotal role in CSC maintenance from head and neck cancers [33]. Together, these date suggest ALDH and CD44 could mediate drug-resistance directly or through stem cell regulatory programs. Indeed, in our study, concomitant CD44 depletion and ALDH inhibition led to sensitization of resistant cells to gefitinib and cisplatin, as well as down-regulation of pluripotency genes *NANOG, SOX2 and POU5F1*.

Other potential mechanisms enhancing tumorigenicity and drug resistance of the ALDH^hi^CD44^hi^ TIC include a higher G2/M fraction, elevated cyclin B1 and B2 expression and correspondingly, a higher population growth rate. This population also showed elevated expression of the double strand break DNA repair gene *RAD51* which is associated with resistance to radiation, chemotherapeutic agents and tyrosine kinase inhibitor therapy [34-36]. Thus, enhanced DNA damage repair and more rapid cell replenishment could facilitate maintenance of the ALDH^hi^CD44^hi^ population. Furthermore, TGFβ-dependent IL6 secretion has been shown to contribute to primary and acquired erlotinib resistance in lung cancer patients [37]. Our data showed ALDH^hi^CD44^hi^ population had higher mRNA expression of the IL6 co-receptor *IL6ST*, suggesting TGFβ-induced resistance could reside mainly in TIC.

Expression profiling studies have shown enrichment of embryonic or tissue stem cell signatures is associated with increased risk of tumor metastasis, poor morphological differentiation and adverse patient outcomes, raising the possibility patient prognosis might be predicted by an appropriate TIC marker. Previous reports on the relevance of ALDH or CD44 single marker have yielded controversial information. For example, ALDH has been reported both as an adverse and a favorable prognostic indicator [21, 38]. For CD44, our previous study has shown a contradictory association with well-differentiated AD and a longer patient survival in lung adenocarcinomas [16]. In this study, there was no statistical evidence of global ALDH and CD44 co-regulated expressions. Notably, when analysis was limited to tumors containing ALDH/CD44-coexpressing cells, those with a higher abundance of TIC was observed to associate with a shorter recurrence free survival (*p* = 0.053). The finding suggested ALDH/CD44 co-expression could be a useful prognostic indicator and supported the relevance of these molecules as lung TIC marker. Some tumors lacked expression of either marker, suggesting other lung TIC indicators remain to be described.

We have shown comprehensive *in vitro, in vivo* data as well as clinical support that the ALDH^hi^CD44^hi^ compartment carries TIC capabilities and is the cellular mediator of drug resistance in lung cancer. These findings are useful in future studies addressing TIC-related cancer biology and development of TIC-targeting therapy.

## MATERIALS AND METHODS

### Cell lines

Established human NSCLC cell lines were obtained from ATCC. PDCL were raised from resected lung cancers or malignant effusions and only 1st to 5th passage cells were used for study. Gefitinib or cisplatin-resistant (-GR or -CR) cells were generated by chronic graded exposure of parental cells to increasing doses of the respective drugs. Cells were maintained in RPMI-1640 (Life Technologies Inc.) or ACL4 with 10% FBS [22].

### Flow cytometry and fluorescence activated cell sorting (FACS)

ALDH activity was analyzed by the Aldefluor kit (Stem Cell Technologies) according to manufacturer's instructions. Briefly, cells were suspended with activated Aldefluor substrate with or without diethylaminobenzaldehyde (DEAB) specific inhibitor as control at 37°C for 30 minutes. CD44 expression was stained by anti-CD44-APC or anti-CD44-PE (BD Pharmingen) as previously described [16]. Cells were fixed and permeabilized (Invitrogen) before antibody incubation for NANOG (Cell signaling), POU5F1 (Chemicon) and SOX2 (Cell signaling) analyses. Corresponding isotype-matched immunoglobulins were used as controls (BD Pharmingen). Non-viable cells were identified by propidium iodide inclusion. Mouse cells were gated out by Pacific blue-labeled anti-mouse CD31 and lineage and H-2K^d^ staining (BioLegend). Flow cytometry was performed using FACSCanto II (BD Biosciences) and figures were produced using FlowJo (Tree star). Cells showing ALDH and CD44 co-expression compared to staining controls were designated as ALDH^+^CD44^+^, those showing no expression of either marker as ALDH^−^CD44^−^, and those showing either marker only as ALDH^+^CD44^−^ or ALDH^−^CD44^+^, respectively. For comparative studies between different ALDH/CD44 populations, cells showing the top/bottom 1 to 5% expression levels were freshly isolated by FACS using BD Aria (BD Biosciences) and designated accordingly as ALDH^hi^CD44^hi^, ALDH^hi^CD44^lo^, ALDH^lo^CD44^hi^ and ALDH^lo^CD44^lo^ populations. Each batch of cells was re-analyzed after collection to ensure a purity of > 90%.

### Sphere formation assay

One thousand freshly isolated cells were cultured in an ultra-low plate (Costar) with serum-free medium containing FGF, EGF and IGF for 21 days as previously described [16].

### Cell invasion assay

Cell invasion assay was performed using transwell® (Costar) coated with matrigel (BD Pharmingen) according to manufacturer's instructions.

### Cell cycle analysis

Freshly sorted cells were fixed in cold 70% ethanol, incubated in PBS buffer containing 50 μg/mL propidium iodide, 100 μg/mL RNAse A and 0.05% Triton X-100 at 37°C for 45 minutes and analyzed using flow cytometry. The proportion of cells in G0/G1, S and G2/M was calculated by FlowJo (Tree star).

### Drug sensitivity and apoptosis assays

Drug sensitivity assay was performed by MTT test and apoptosis was quantified by Annexin V-FITC and PI staining as previously described [16].

### Gene expression analysis

Gene mRNA expression was analyzed by quantitative RT-PCR (QPCR) (7900HT, Applied Biosystems) and SYBR green detection. Expressions of *GAPDH* and *beta-2-microglobulin* (*B2M*) were averaged and used as internal controls. Primers were listed in Supplementary Table S5.

### *In vivo* tumorigenicity and TKI response

All animal experiments were approved and performed according to guidelines by the Animal Ethics Committee, the University of Hong Kong. Briefly, freshly sorted cells mixed with an equal volume of matrigel (BD Pharmingen) were injected subcutaneously at the back of 6-week severe combined immunodeficiency (SCID) mice. For serial transplantation, the previous generation xenografts derived from the respective ALDH/CD44 subsets were digested to obtain single cell suspensions, stained, subjected to FACS to isolate the parental subset and transplanted into subsequent recipients. To investigate the *in vivo* TKI response, mice bearing xenografts from HCC827 were subjected to intraperitoneal gefitinib (Selleck) injections at 50 mg/kg in 0.2 mL of 1% Tween 80 five times per week for 4-5 weeks [39, 40]. Gefitinib was dissolved in DMSO; a mixture of 1% Tween 80 with an equal volume of DMSO was used as vehicle control.

### CD44 knockdown, ALDH, Hedgehog and Notch pathways inhibition

Small interfering RNA (siRNA) targeting human CD44 and non-targeting siRNA (Qiagen) were used for CD44 knockdown. Transfection was performed using Genemute (SignaGen Laboratories) according to the manufacturer's instructions. DEAB (Sigma) was constituted in 100% ethanol and diluted in media to 100 μM. The ALDEFLUOR Assay was used in conjunction with flow cytometry to assess ALDH activity following DEAB treatment. Cyclopamine (Selleck) or RO4929097 (Selleck) was used for inhibition of Hedgehog or Notch pathway, respectively.

### Immunohistochemistry (IHC) for ALDH and CD44 expression in clinical lung cancers

Formalin-fixed paraffin-embedded tumor tissues from 193 surgically resected primary lung carcinomas without neoadjuvent therapy were arrayed on tissue micro-array (TMA) with each case represented by 3 to 5 tumor cores. Tissues from fetal lung, reactive pulmonary lesions and a representative lung cancer were included in each TMA block for standardization. IHC for CD44 and ALDH were performed separately using anti-CD44 standard form (clone 156-3C11, 1:200 dilution, BiocareMedical) or anti-ALDH1A1 (1:1000 dilution, Abcam) as previously described [16]. Primary antibodies were replaced by SignalStain® Antibody Diluent (Cell Signaling Technology) in the negative controls. Expression levels were semi-quantitatively analyzed using an automated image capturing and analysis system (Aperio) [16]. Briefly, representative tumor areas in each tissue core excluding stroma, necrosis or inflammatory regions were gated on the scanned images. The staining intensities and cell abundance in each annotated area were computed using standard algorithms. Data from all tissue cores were integrated and a single score reflecting the abundance of ALDH or CD44-expressing cells, respectively, was assigned to each case. For ALDH/CD44-coexpresssion grading, only co-localized tumor cells with simultaneous ALDH and CD44 staining were counted. Tumor typing and pathological staging were according to W.H.O. criteria [41]. Patient outcomes were collated by the clinician in-charge.

### Statistical methods

Differences between groups were analyzed by student's *t* test for continuous variables. Correlation between clinicopathological variables and ALDH or CD44 expression were analyzed by χ2 test or Fisher Exact tests. Differences in patient survival were analyzed by log rank test and survival curves were drawn by Kaplan Meier method. Independent prognostic indicators for survival were analyzed by the Cox regression model. All statistical tests were analyzed by SPSS 18 for Windows (SPSS Inc., Chicago, IL). A 2-tailed *p* value of < 0.05 was considered as the threshold for statistical significance.

## Supplementary Figures and Table


