# Supplementary material for: Lung cancer tumorigenicity and drug resistance are maintained through ALDHhiCD44hi tumor initiating cells
Source: Oncotarget. 2013 Aug 30;4(10):1698–711. doi: 10.18632/oncotarget.1246 (PMC3858556; doi:10.18632/oncotarget.1246)
Supplement: Supplementary file 1 [file oncotarget-04-1698-s001.docx]

**Supplementary Tables and Figure**

**Supplementary Table S1. Distribution of ALDH/CD44 subsets in NSCLC cell lines**

| Cell lines | ALDH^-^CD44^+^ | ALDH^-^CD44^-^ | ALDH^+^CD44^+^ | ALDH^+^CD44^-^ |
| --- | --- | --- | --- | --- |
| H1650 | 43.14 | 48.62 | **2.41** | 4.60 |
| HCC827 | 15.67 | 65.08 | **5.87** | 15.27 |
| H1299 | 58.10 | 32.50 | **6.86** | 2.55 |
| HCC827 GR 5µM | 32 | 31.9 | **23.3** | 12.8 |
| HCC827 GR 10µM | 8.83 | 28.2 | **35.98** | 27 |
| Patient #2* | 3.19 | 68.20 | **3.39** | 25.20 |
| Patient #10* | 2.27 | 88.40 | **1.24** | 8.13 |
| Patient #18* | 41.80 | 56.40 | **0.75** | 1.01 |
| Patient #20* | 52.2 | 28.10 | **12.40** | 7.67 |
| HKULC3* | 12.50 | 82.20 | **2.22** | 3.14 |
| Patient #24* | 87.70 | 1.96 | **9.94** | 0.35 |

*, Patient-derived cancer cell lines established from local patients. Results represent average of three individual flow cytometry experiments.

**Supplementary Table S2. *In vivo* 1^st^ generation tumorigenecity of ALDH/CD44 subsets**

| Cell subset | | 100,000 cells | | 500 cells | |
| --- | --- | --- | --- | --- | --- |
|  |  | tumor incidence | Latency (days) | tumor incidence | Latency (days) |
| H1650 | Unsorted | 4/6 | 32.5 ± 5.0 | 0/3 | N/A |
|  | ALDH^lo^CD44^lo^ | 2/6 | 70.0 ± 8.0 | 0/3 | N/A |
|  | ALDH^lo^CD44^hi^ | 4/6 | 30.0 ± 2.1 | 0/3 | N/A |
|  | ALDH^hi^CD44^lo^ | 4/4 | 34.3 ± 4.8 | 0/3 | N/A |
|  | ALDH^hi^CD44^hi^ | 6/6 | 20.3 ± 0.2 | 2/3 | 110.0 ± 5.1 |
| HCC827 | Unsorted | N/A | N/A | 0/3 | N/A |
|  | ALDH^lo^CD44^lo^ | N/A | N/A | 0/3 | N/A |
|  | ALDH^lo^CD44^hi^ | N/A | N/A | 0/3 | N/A |
|  | ALDH^hi^CD44^lo^ | N/A | N/A | 0/3 | N/A |
|  | ALDH^hi^CD44^hi^ | N/A | N/A | 3/3 | 52.7 ± 9.0 |

N/A, tumor dimension not available

**Supplementary Table S3. Comparison of ALDH^hi^ single or ALDH^hi^ /CD44^hi^ double marker tumorigenicity in SCID mice**

|  | ALDH^hi^ | | | | ALDH^hi^CD44^hi^ | | | |
| --- | --- | --- | --- | --- | --- | --- | --- | --- |
|  | 2,500 cells | | 10,000 cells | | 2,500 cells | | 10,000 cells | |
|  | Tumor  incidence | Latency (days) | Tumor  incidence | Latency (days) | Tumor  incidence | Latency (days) | Tumor  incidence | Latency (days) |
| H1650 | 4/6 | 55 | 3/3 | 45 | 6/6 | 41 | 6/6 | 20.3 |
| HCC827 | 3/7 | 70 | 3/3 | 55 | 3/3 | 29 | N/A | N/A |

N/A, tumor dimension not available

**Supplementary Table S4. Summary of ALDH and CD44 expression in clinical lung cancers**

| **Clinical-Pathological Variables** | **No. of cases (n=193)** | **ALDH expression**  **Number of cases (%)** | | | **CD44 expression**  **Number of cases (%)** | | |
| --- | --- | --- | --- | --- | --- | --- | --- |
|  |  | **None*** | **Yes#** | **p** | **None*** | **Yes#** | **p** |
| **Gender** |  |  |  |  |  |  |  |
| M | 119 | 67 (56.3) | 52 (43.7) |  | 45 (37.8) | 74 (62.2) |  |
| F | 74 | 44 (59.5) | 30 (40.5) | 0.765 | 29 (39.2) | 45 (60.8) | 0.880 |
| **Smoking Hx** |  |  |  |  |  |  |  |
| Never smoker | 87 | 51 (58.6) | 36 (41.4) |  | 31 (35.6) | 56 (64.4) |  |
| Tobacco-exposed | 106 | 60 (56.6) | 46 (43.4) | 0.884 | 43 (40.6) | 63 (59.4) | 0.552 |
| **Tumor Type** |  |  |  |  |  |  |  |
| Adenocarcinoma | 140 | 87 (62.1) | 53 (37.9) |  | 56 (40.0) | 84 (60.0) |  |
| SCC | 27 | 9 (33.3) | 18 (66.7) | **0.006** | 5 (18.5) | 22 (81.5) | **0.048** |
| Others^@^ | 26 | 15 (57.7) | 11 (42.3) | **0.021** | 13 (50.0) | 13 (50.0) | **0.046** |
| **Differentiation Status** |  |  |  |  |  |  |  |
| WD | 47 | 26 (55.3) | 21 (44.7) |  | 12 (25.5) | 35 (74.5) |  |
| MD | 81 | 42 (51.9) | 39 (48.1) |  | 32 (39.5) | 49 (60.5) |  |
| PD | 65 | 43 (66.2) | 22 (33.8) | 0.208 | 30 (46.2) | 35 (53.8) | 0.083 |
| **Pathological Stage** |  |  |  |  |  |  |  |
| I | 128 | 73 (57.0) | 55 (43.0) |  | 49 (38.3) | 79 (61.7) |  |
| II | 37 | 17 (45.9) | 20 (54.1) |  | 14 (37.8) | 23 (62.2) |  |
| III | 28 | 21 (75.0) | 7 (25.0) | 0.063 | 11 (39.3) | 17 (60.7) | 0.993 |
| **ALDH expression** |  |  |  |  |  |  |  |
| No expression | 111 | - | - |  | 43 (38.7) | 68 (61.3) |  |
| Low abundance | 24 | - | - |  | 8 (33.3) | 16 (66.7) |  |
| High abundance | 55 | - | - | - | 23 (41.8) | 32 (58.2) | 0.859 |
| **CD44 expression** |  |  |  |  |  |  |  |
| No expression | 74 | 43 (58.1) | 31 (41.9) |  | - | - |  |
| Low abundance | 33 | 21 (63.6) | 12 (36.4) |  | - | - |  |
| High abundance | 86 | 47 (54.7) | 39 (45.3) | 0.669 | - | - | - |

*, tumors showing no expression of the respective marker; #, tumors showing expression of the respective marker in ≥1 tumor cell; SCC, squamous cell carcinoma; @, incuded 10 large cell, 5 adenosquamous, 2 mucoepidermoid, 4 high grade neuroendocrine and 5 lymphoepithelioma-like carcinomas; WD, well differentiated; MD, moderately differentiated; PD, poorly differentiated.

**Supplementary Table S5. List of Q-PCR primers**

| Gene | Primer sequences |
| --- | --- |
| *NANOG* | F: AAGGTCCCGGTCAAGAAACAG  R: CTTCTGCGTCACACCATTGC |
| *SOX2* | F: GCCGAGTGGAAACTTTTGTCG  R: GGCAGCGTGTACTTATCCTTCT |
| *POU5F1* | F: GGCAACCTGGAGAATTTGTT  R: GTGCATAGTCGCTGCTTGAT |
| *BMI1* | F: GATGGCCGCTTGGCTCGCAT  R: GTACCCTCCACAAAGCACACACAT |
| *CDH1* | F: AAAGGCCCATTTCCTAAAAACCT  R: TGCGTTCTCTATCCAGAGGCT |
| *VIM* | F: GGAAGCCGAAAACACCCTG  R: GAGACGCATTGTCAACATCCT |
| *TWIST1* | F: GCAGGGCCGGAGACCTAGATGTC  R: CCGCTGCCCGTCTGGGAATC |
| *ZEB1* | F: TTACACCTTTGCATACAGAACCC  R: TTTACGATTACACCCAGACTGC |
| *SNAI2* | F: AAGCATTTCAACGCCTCCAAA  R: GGATCTCTGGTTGTGGTATGACA |
| *CCNB1* | F: TTGGGGACATTGGTAACAAAGTC  R: ATAGGCTCAGGCGAAAGTTTTT |
| *CCNB2* | F: CCGACGGTGTCCAGTGATTT  R: TGTTGTTTTGGTGGGTTGAACT |
| *RAD51* | F: CGAGCGTTCAACACAGACCA  R: GTGGCACTGTCTACAATAAGCA |
| *IL6ST* | F: CGGACAGCTTGAACAGAATGT  R: ACCATCCCACTCACACCTCA |
| *CD44* | F: TCCAACACCTCCCAGTATGACA  R: GGCAGGTCTGTGACTGATGTACA |
| *GAPDH* | F: GAGTCAACGGATTTGGTCGTAT  R: ATGGGTGGAATCATATTGGAAC |
| *B2M* | F: AGG CTA TCC AGC GTA CTC CA  R: GGC ATC TTC AAA CCT CCA T |


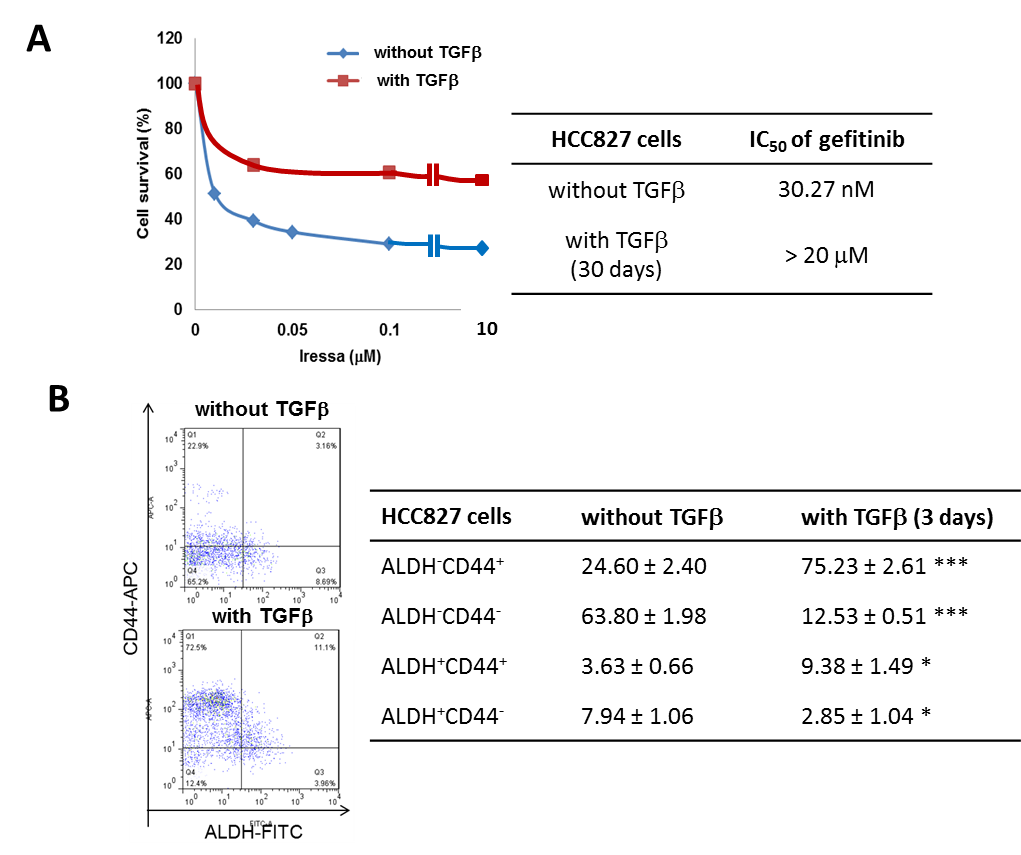


**Supplementary Figure S1. TGFβ** **induced increased ALDH^+^CD44^+^ subset in HCC827 cells.** A, TGFβ treatment caused reduced sensitivity to gefitinib (iressa) in HCC827 cells. B, TGFβ treatment increased HCC827 ALDH^+^CD44^+^ while decreased ALDH^-^CD44^-^ cells by flow cytometry. *, *p* < 0.05; **, *p* < 0.01; ***, *p* < 0.001, compared with control. Data represent mean ± SD of triplicate experiments.
